# A population-based repeated cross-sectional study using administrative health data to examine the impact of the COVID-19 pandemic on mental wellness in citizens of the Métis Nation of Ontario

**DOI:** 10.23889/ijpds.v9i5.2631

**Published:** 2024-09-18

**Authors:** Abigail Simms, Tera Beaulieu, Benny Michaud, Noel Tsui, Brandon Zagorski, Sarah Edwards, Maria Chiu

**Affiliations:** 1 ICES; 2 Métis Nation of Ontario; 3 University of Toronto

## Objective and Approach

The COVID-19 pandemic led to unprecedented levels of mental unwellness and yet there are no reports to date that share Métis-specific data. Our study examined changes in patterns of mental and addictions-related (MHA) outpatient health service utilization using population-based data on Métis Nation of Ontario (MNO) citizens before and during the COVID-19 pandemic. Administrative health data in Ontario, Canada between 2017 and 2022 was linked with the MNO's Citizenship Registry (2022) under an existing Data Governance and Sharing Agreement. Monthly rates of MHA outpatient visits were compared between the pre-COVID-19 period (March 2017 to February 2020) and post-COVID-19 onset (March 2020 to December 2022), and rate ratios comparing observed and expected rates were derived using Poisson generalized estimating equations. Stratifications by age and sex were examined.

## Results and Conclusion

Among 28,400 MNO citizens (50.3% male; median age 44 years; 28.3% living rurally), the average monthly rate of MHA outpatient visits was 47.4 per 1000 population pre-COVID-19. During the COVID-19 pandemic, the observed MHA outpatient visit rates were 13% higher than expected (RR=1.13; 95% CI: 1.02-1.26). MHA outpatient visits were higher in females compared to males and MNO citizens aged 30 to 64 years versus older or younger citizens.

## Implications

This study is the first to use population-level study to examine MHA health service use in Métis people. High-quality, contemporary data on the mental health outcomes of MNO citizens is crucial to inform MNO’s Mental Health and Addictions programming, resource allocation and emergency preparedness.

